# Proceedings of the 2025 NASSi Annual Meeting

**DOI:** 10.1016/j.xnsj.2025.100754

**Published:** 2025-07-21

**Authors:** Patrick C. Hsieh, Chiung-Chyi Shen, Alan S. Hilibrand, D. Scott Kreiner, David R. O’Brien, Hani H. Mhaidli, Jau-Ching Wu, Wen-Cheng Huang

**Affiliations:** a Department of Neurological Surgery, University of Southern California, Keck School of Medicine, USC Spine Center, Los Angeles, California, United States; b Department of Neurosurgery, Taichung Veterans General Hospital, Taichung, Taiwan; c Department of Orthopedic Surgery, Rothman Institute, Sidney Kimmel Medical College at Thomas Jefferson University, Philadelphia, Pennsylvania, United States; d Barrows Neurological Institute, Phoenix, Arizona, United States; e Department of Orthopaedics, Rehabilitation, and Department of Neurosurgery, Wake Forest University School of Medicine, Winston-Salem, North Carolina, United States; f Spine Department at the Hospital Universitario de Gran Canaria, Las Palmas, Spain; g Department of Neurosurgery, Neurological Institute, Taipei Veterans General Hospital, Taipei, Taiwan

***2025 NASS International Meeting: Message from the chairs***

The North American Spine Society (NASS) International Committee is pleased to host the 2025 NASS International Meeting in Taipei, Taiwan, from July 21 to 26, 2025. We are proud to co-host this event with our esteemed colleagues from the Taiwan Neurosurgical Society (TNS), the Taiwan Neurosurgical Spine Society (TSNS), and the Taiwan Society of Minimally Invasive Spine Surgery (TSMISS). The presented proceedings highlight the scientific accomplishments that will be presented at this meeting.

This conference builds on the success of the inaugural NASS International Meeting in 2023, held in Bangkok, Thailand, which drew over 16 international societies and attendees from more than 30 countries. We are thrilled to present another exceptional conference in Taipei this July. The 2025 NASS International Meeting features over 20 international societies, offering a diverse range of activities that include hands-on courses, research presentations, symposia, and keynote lectures.

Our mission at NASS International is to advance spine care globally through improved education, increased access to educational resources, and fostering international collaboration among NASS International members and partner societies. The NASS International meeting is a key initiative developed in partnership with our international members and partner societies. The global community has strongly recommended that NASS host high-quality meetings in regions closer to its international members, facilitating opportunities for partnerships among these members and societies in NASS's educational events. NASS International is thrilled to bring this vision to life for our international members and partner societies.

The 2025 NASS International Meeting in Taipei, Taiwan, promises to be an exciting event that showcases the latest research, advancements in spine care, and innovations in spine surgery techniques and technology from around the world. The combination of abstract presentations and expert lectures will cover a wide breadth of spine topics and represent the various international societies participating in the event.

The abstracts included here emphasis the focus on minimally invasive surgery (MIS) and endoscopic spine surgery. A notable study will analyze risk factors for reoperation after minimally invasive transforaminal interbody fusion in nearly 800 patients, while another will compare mini-open TLIF versus hybrid endoscopic TLIF techniques. Artificial intelligence (AI) is at the forefront of today’s technological advancements, and its importance for the future of spine surgery and medicine cannot be overstated. An exciting study will demonstrate how deep learning can automate the Lenke classification using anatomical landmarks from X-rays. Additionally, a presentation will discuss the development of a machine learning model designed to predict adjacent segment fractures following cementoplasty, highlighting the significant potential impact of AI in the future spine.

Additionally, the abstracts highlight research on basic science topics, including the effects of duration and compression on dorsal root ganglia in chronic pain, implant material studies in rabbits, and predictive analytics for pedicle screw loosening using MRI imaging. Overall, the content of the conference showcases significant advancements in spine surgery and multidisciplinary care.

We are excited that NASSJ is publishing the proceedings from this event. We are grateful to have the opportunity to document its contents and share the abstracts with all interested readers. It is hoped that these proceedings will be useful to those attending the meeting in Taipei, Taiwan, and provide a window into the content of the meeting for those who cannot attend. A heartfelt thank you to the NASSJ team for making this possible!

## Declarations of competing interest

The authors declare that they have no known competing financial interests or personal relationships that could have appeared to influence the work reported in this paper.

